# Hacking techniques improve health and nutritional status of nestling White‐tailed Eagles

**DOI:** 10.1002/ece3.9776

**Published:** 2023-02-09

**Authors:** Miguel Ferrer, Rhian Evans, Joanna Hedley, Simon Hollamby, Anna Meredith, Virginia Morandini, Owen Selly, Claire Smith, D. Philip Whitfield

**Affiliations:** ^1^ Applied Ecology Group Estación Biológica de Doñana (CSIC) Seville Spain; ^2^ The Royal Society for the Protection of Birds Edinburgh UK; ^3^ The Royal (Dick) School of Veterinary Services and the Roslin Institute University of Edinburgh Edinburgh UK; ^4^ The Royal Veterinary College London UK; ^5^ Territory Wildlife Park Berry Springs Northern Territory Australia; ^6^ Veterinary Department Perth Zoo South Perth Western Australia Australia; ^7^ Melbourne Veterinary School University of Melbourne Melbourne Victoria Australia; ^8^ Oregon Cooperative Fish and Wildlife Research Unit, Department of Fisheries and Wildlife Oregon State University Corvallis Oregon USA; ^9^ Natural Research Ltd. Banchory UK

**Keywords:** blood parameters, cholesterol, free‐living raptor, hatching date, hematology, plasma biochemistry, urea

## Abstract

Birds of prey frequently feature in reintroductions and the hacking technique is typically used. Hacking involves removing large nestlings from donor populations, transferring them to captivity, feeding them ad libitum. Potentially, via the hacking method, the stress of captivity and disruption of parental feeding may be detrimental. Alternatively, the provision of ad libitum food may be advantageous. Although hacking has underpinned reintroduction project successes there has been no research on how the method may affect the health and nutritional status of translocated birds during captivity. We compared blood chemistry data from 55 young White‐tailed Eagles, translocated from Norway as part of the species' reintroduction to Scotland, from sampling soon after arriving in captivity and again (≈42 days later) before their release. Numerous significant differences between the first and second samples were found, but no significant interactions showed that the sexes responded similarly to captivity. According to hematological and biochemical metrics, individuals showed several changes during captivity, including in red blood cell parameters, plasma proteins, and white cellular parameters related to the immune system, that indicated improved health status. Captivity with ad libitum food was associated with decreased urea and uric acid values: high values can indicate nutritional stress. Urea values became more normally distributed before release, indicating that ad libitum food had reduced nutritional differences between early nestlings in the season and later ones. Despite plentiful food, both sexes lost body mass before release, suggesting an inherent physiological mechanism to improve flight performance in fledglings. We conclude that hacking improved the health and nutritional status of released eagles which is likely to enable birds to cope with greater costs of exploratory behavior which they may require in reintroduction projects. In this context, we note the absence of survival differences between hacked and wild raptors in previous research.

## Graphical abstract

Hacking techniques used with the White‐tailed eagles improved nutritional and health status of nestlings, increasing their survival possibilities during dispersal.

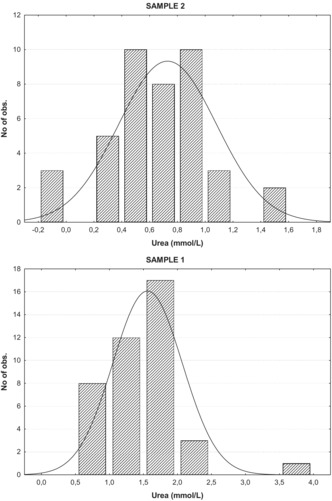

## 1 INTRODUCTION

The global loss of biodiversity is well‐documented, with increasing numbers of species at risk of extinction due to direct or indirect anthropogenic causes (Mora et al., 2013; Seddon et al., 2014). Management to reduce the risk of species' extinction includes a wide variety of actions, including reintroduction. Reintroductions are intentional translocations of species into parts of their historically known range from which they have been extirpated (IUCN, 2013). Wildlife reintroductions are becoming increasingly common, being now considered an important tool for the conservation of endangered or threatened species (Armstrong & Seddon, 2008; Soorae, 2016).

Birds of prey (raptors and vultures) are frequently involved in reintroduction programs. Several such programs have been successful and typically involve a common practical method: ‘hacking’ (Evans et al., 2009; Ferrer et al., 2018). This method involves taking large nestlings (typically when around two‐thirds toward fledging in growth) from a robust donor population and transferring them to the incipient novel population via hacking facilities. Usually, donor nestlings are translocated to artificial nest(s) inside hacking facilities (effectively large aviaries) situated geographically in the recipient area for the new population. During this period, translocated birds are fed ad libitum until they are released from aviaries once it is judged that the birds are capable of free flight (effectively simulating ‘natural’ fledging age: Muriel et al., 2011).

Reintroductions by means of the hacking method appear especially effective in birds of prey (Cade, 2000; Evans et al., 2009; Ferrer & Morandini, 2017; Morandini & Ferrer, 2017a) and therefore can be used as potentially useful tools for population restoration with appropriate planning, development, and monitoring (IUCN, 2013; Morandini & Ferrer, 2017b). There is evidence that in some species at least, manipulation associated with hacking (extraction of the nestlings, ringing and tagging them, unlimited amounts of food, keeping them in captivity for some period, absence of parents) seems to have nonharmful effects on survival, subsequent reproduction, habitat selection or dispersive behavior (Ferrer & Morandini, 2017; Morandini et al., 2017, 2019; Morandini & Ferrer, 2017a; Muriel et al., 2015, 2016, 2020). Nevertheless, as far as we know there are no studies on potential consequences of health and nutritional condition due to the hacking method, where food is provided ad libitum and there is no contact with the parents after translocation.

Avian blood chemistry and blood cell analyses can detect possible pathological states (Meredith et al., 2012). An adequate knowledge of hematological values is recommended for projects involving research and management of populations since they can be valuable for the assessment of the nutritional levels and health status of constituent individuals (Ferrer & Dobado‐Berrios, 1998; Ferrer et al., 2017; Meredith et al., 2012). Despite the technology to analyze concentrations of blood constituents being widely available and well understood, studies of blood parameters in free‐living raptors are still few (Dobado‐Berrios & Ferrer, 1997; Ferrer & Dobado‐Berrios, 1998; Flo et al., 2019; Hernández & Margalida, 2010; Meredith et al., 2012; Montolio et al., 2018; Viñuela et al., 1991). Hematological values, including chemical components, are known to be influenced by many factors: physiological state, age, sex, nutritional condition, circadian rhythm, seasonal changes, captivity, pollutants, and plasma‐storing methods (Ferrer et al., 1987; García‐Rodriguez, Ferrer, Carrillo, & Castroviejo, 1987; García‐Rodriguez, Ferrer, Recio, & Castroviejo, 1987; Gee et al., 1981; Jenni‐Eiermann & Jenni, 1992; Rehder & Bird, 1983; Viñuela et al., 1991).

Translocations and reintroductions seem likely to be used more extensively in the future, especially in the face of rapid global changes and corresponding distributional shifts of certain species, but also with regard to the favorable socio‐ecological conditions that remain within the former ranges of threatened species. Consequently, it is important to improve our understanding of the limitations and applications of associated techniques, sharing results and thus increasing our expertise in wildlife restoration strategies.

The aim of this study was to analyze the potential effect of hacking on health and nutritional status of White‐tailed Eagle (WTE: *Haliaaetus albicilla*) nestlings, in over 50 birds translocated from western Norway to eastern Scotland. Blood samples were taken at two events and subsequently analyzed using hematological and biochemical metrics. The first sample was obtained when birds had been transferred to Scotland soon after translocation, and the second after birds had been held in captivity and fed ad libitum for c. 44 days shortly before their release from captivity. We used this double sampling (including biometric data) across the hacking period, to examine if the period of captivity had caused any deterioration in health or nutritional status. Alternatively, the hacking method may have been beneficial with ad libitum food improving the health and nutritional status of translocated birds during captivity.

## 2 MATERIALS AND METHODS

### 2.1 Study species and area

White‐tailed Eagle is a large bird of prey with females being larger than males. The species is Eurasian in distribution and ecologically the Old World equivalent of the New World's Bald Eagle (*Haliaeetus leucocephalus*). Our study was involved with the second phase (east Scotland) of a reintroduction initiative instigated by an initial (1975–1985: 82 birds) reintroduction and later reinforcement translocations (1993–1995: 59 birds) using birds from western Norway to the west coast of Scotland (Evans et al., 2009; Green et al., 1996; Sansom et al., 2016; Whitfield, Douse, et al., 2009; Whitfield, Duffy, et al., 2009). The core initiative allowing our study aimed to provide a further nucleus of WTE population growth on the east coast of Scotland, situated away from the previous centers of population reestablishment and subsequent expansions on the west coast.

We sampled nestlings extracted from a wild donor WTE population in Norway: every June between 2007 and 2012 WTE nestlings were collected from monitored nests in the western counties of Møre og Romsdal, Hordaland and Sogn og Fjordane. Single birds were extracted from nests that had two or three chicks which from field observations were deemed old enough (5–8 weeks old) to be suitable for translocation on plumage features and physical appearance (Helander, 1981). Extractions were coordinated with the goal of a balanced sex ratio (based on biometrics). Birds were uniquely metal banded (BTO: British Trust for Ornithology) in Norway prior to translocation, for subsequent identification.

There were extractions of 15 (2007, 2008, 2009), 19 (2010), 16 (2011), and 6 (2012) nestlings per annum, resulting in 86 translocated birds (85 were released as one 2009 bird died in captivity from aspergillosis, despite veterinary intervention). Of these, 55 were double sampled for biochemical and hematological parameters 2009–2012 and are the subject of this study.

After transport from Norway, all birds had a full clinical examination within 24 h of arrival to identify any potential abnormalities. Translocated birds were kept at a hacking facility in northeast Fife, southeast Scotland, in wooden (three sides enclosed) and wire mesh aviaries measuring approximately 3.6 × 3 × 2.7 m that had ‘nesting’ platforms covered in bark chips and soft vegetation, and at least two long perches. Two or three birds were housed in each aviary, with similarly sized birds being kept together. Birds were supplied with ad libitum food involving the wide range exploited by WTE: fish, birds and mammals, with vitamin and mineral supplements (Nutrobal®; Vertak) added to the food daily. Contact with humans was minimized by food being provided via a hatch in the aviaries' back panel. Captive eagles were kept under daily observation via ‘spy holes’ in aviaries' back panels for surveillance of their status and any signs of injury or ill‐health, with a qualified veterinarian on call should the need arise: no eagles were injured because of our activities.

The first sampling was undertaken shortly after all birds were in the hacking facility. This allowed for greater efficiency in sampling all birds at the same time after extraction and transportation from Norway to Scotland (Table 1). The second sampling was taken shortly before birds' release from captivity when they were also fitted with patagial wing tags or color‐bands and a VHF telemetry tag, when biometric data (including body mass) were again taken (Table 1). Birds were aged (days since hatch date) using the method of Løseth et al. (2019): after T. Nygård (personal communication) involving measurement of a central tail feather. This method was preferred to those in Helander (1981) and Helander et al. (2007) involving other recorded biometrics when like Løseth et al. (2019) we also found the “Nygård” method to produce more realistic and consistent results.

**TABLE 1:** Summary by year of the median date (range) when birds were collected from Norway, the sexes of the collected birds, the dates when first and second samples were taken in Scotland, and median date (range) when birds were released from the hacking aviaries.

Year	Median collection date (range)	Male	Female	First sample date	Second sample date	Median release date (range)
2009	June 19 (June 13–25)	7	7	June 26	August 7	August 12 (August 11–25)
2010	June 18 (June 10–24)	7	12	June 25	August 11	August 17 (August 16–19)
2011	June 18 (June 13–22)	10	6	June 24	August 5	August 11 (August 8–19)
2012	June 20 (June 14–22)	2	4	June 22	August 10	August 21 (August 20–23)

*Note*: See main text for details on samples taken on first and second dates, and sexing method.

### 2.2 Blood collecting procedures and sex determination

The first blood samples were drawn from nestlings 41–66 days (mean = 52.4, SD = 5.7) after their estimated hatch, corresponding to the beginning of hacking. The second samples were drawn when nestlings were between 86–108 days old (mean = 97.6, SD = 5.5), just prior to their release (Table 1). A total of 55 nestlings, 26 males and 29 females, were double sampled. Intervals between repeat samples of the same individual were between 42–47 days (mean = 44.0, SD = 2.0).

Blood samples were collected between 11:00 a.m. and 3:00 p.m. to avoid variations in blood parameters due to circadian rhythms (Ferrer, 1990; Ferrer et al., 1994; García‐Rodriguez, Ferrer, Recio, & Castroviejo, 1987). Blood samples were collected from the cutaneous ulnar (brachial) vein with the birds cast in dorsal recumbency. Total blood collected in each extraction was 2 mL and range of weight of the nestlings was 2500–5600 g. Blood was collected in a 10 mL plastic syringe (Becton Dickinson S.A.) attached to a 23 gauge 1″ needle (Monoject). Three blood smears were made using a slide‐on‐slide technique. In total, 1 mL of blood was transferred to a di‐potassium ethylene diamine tetra acetate (EDTA) anticoagulant tube (Teklab) for ‘DNA’ sexing. Birds were subsequently sexed at a (DNA) molecular level. An additional 1 mL EDTA tube was filled and submitted for hematological analysis. A 1 mL tube containing lithium heparin (Teklab) as an anticoagulant was filled for blood lead analysis. The remaining sample was transferred to a 6 mL lithium heparin tube (Becton Dickinson S.A.) and submitted for biochemical analyses. All samples were kept in a cooler box at approximately 4°C for a maximum of 8 h prior to submission for laboratory analysis. Samples arrived within 24 h at Greendale Veterinary Diagnostics, Surrey, UK, where biochemical and hematologic analyses were performed. Blood lead analysis was performed at Veterinary Laboratory Agencies (VLAs). Packed cell volumes and total plasma protein levels were also examined within 3 h of collection at the Veterinary Centre, University of Edinburgh, UK.

### 2.3 Hematological analyses

Red blood cell (RBC) counts were measured using an impedance counter with a floating threshold by a CellTac analyzer (model MEK 5108 K; Nihon Kohden Corporation). Hemoglobin (Hb) was measured photometrically with the HemoCue analyzer (HemoCue, Prospect Diagnostics) with microcuvettes preloaded with reagent. Packed cell volume (PCV) was obtained with a hematocentrifuge by using plain capillary tubes sealed with Critoseal (Krackeler Scientific Inc.). Samples were centrifuged at 10,000 *g* for 5 min and read manually with a hematocrit reader. White blood cell (WBC) counts were determined manually by mixing 0.38 mL of 1% ammonium oxalate with 0.02 mL of EDTA blood and placing this on a rotor for approximately 5 min. The resultant mixture was used to fill the counting chamber of an improved Neubauer hemocytometer and allowed to stand for 5 min in a moist box to allow the cells to settle. Counts were performed with phase contrast microscopy with the 340/0.65 phase objective. A total of four large squares (64 small squares) was counted, and the total number was divided by 20 to obtain WBC × 10^9^/L. Blood smears were stained with May‐Grunwald‐Giemsa. The differential count was based on 100 counted leucocytes. At the same time, thrombocytes were counted manually. For all samples, thrombocytes were estimated to be adequate and none were assessed as having a thrombocytopenia, and so were not considered further.

### 2.4 Biochemical analyses

Samples in lithium heparin were centrifuged at 10,000 *g* for 10 min on arrival at the laboratory and the plasma was separated for biochemical analysis. Plasma was stored frozen at −24°C until analysis. Determinations of 13 parameters were made in sampled birds: albumin (Bromocresol green method), globulin (Biuret method), urea (Urease–GLDH method), glucose (GOD‐PAP method), cholesterol (Chol. esterase chol. oxidase–Trinder method), calcium (o‐cresolphthalein complexone reaction), aspartate aminotransferase AST (IFCC technique), creatinine kinase CK (IFCC technique), uric acid (Uricase–Trinder method), lactate dehydrogenase LDH (Pyruvate–lactate method), sodium (Indirect ion selective electrode), and potassium (Indirect ion selective electrode).

All the biochemical analyses were conducted under ILAB 600 (Instrumentation Laboratory) according to the manufacturer instructions. For some samples, there was insufficient sample volume to realize analysis of all the biochemical metrics. The laboratory's quality control was to run daily internal quality control material and to peer review the results with other laboratories by using two external quality assurance schemes: The Randox International Quality Assessment Scheme and the American Veterinary Laboratory Association.

### 2.5 Statistical analyses

All data are expressed as mean ± standard deviation (SD). Normality in distribution of variables was univariate tested with graphical methods and homogeneity of variances using the Leven test. Some of them were log or square transformed when necessary to meet normality (lymphocytes, monocytes, basophiles, thrombocytes, and cholesterol) after they were back‐transformed for tables and graphics. We used MANOVA and regression methods to examine our hypotheses on blood hematological and biochemistry parameters according to sample timing and birds' age and sex. In analyses, we standardized hatching dates using the earliest estimated record of hatching each year as day 1 and the difference between the earliest record and each subsequent hatch date. Statistica 11.0 software statistical package was used to perform statistical procedures, and we used an *α* value of .05 to assess significance of results.

## 3 RESULTS

**FIGURE 1:**
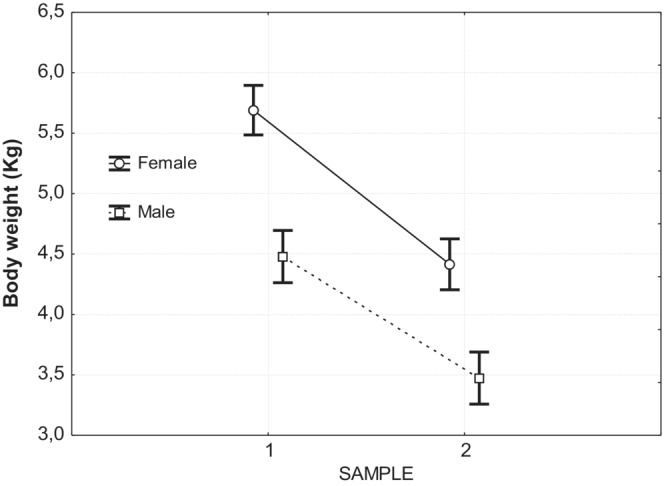
Weight recession of young eagles during hacking documented by the two sampling events. Both sexes lost mass in a similar way (female, upper: male, lower). Circles (female) and squares (males) indicate means and vertical bars denote 0.95 confidence intervals.

Mean values of selected blood parameters according to sample timing are shown in Table 2. Using a MANOVA analysis, significant differences between first and second samples and between sexes were found, but there was no significant interactive effect between sample and sex (Table 3). Significant differences between the two samples were found in most of the parameters (Table 2). Among hematological parameters, all showed significant differences early and later in hacking, including red blood cells, lymphocytes, and monocytes. Hemoglobin, PCV and thrombocytes decreased during captivity, but white blood cells and most related component metric values increased. All the biochemical parameters, apart from AST and LDH, showed significant differences (Table 2). Albumin and globulin increased as did cholesterol, calcium, and potassium. Nitrogenous components, urea or uric acid, decreased together with glucose and sodium. Weights (body mass) of the nestlings varied significantly, being lower in the second sample. This decrease in body mass was similar for both sexes (Figure 1).

**TABLE 2:** Results of MANOVA analysis of body weight, blood chemistry and hematological parameters for WTE nestlings, when entering the hacking facility (52 ± 5 days of age), and after 44 ± 2 days with ad libitum food, when they were 97 ± 5 days old, a few days before their release from the hacking facility.

Parameter	*n*	Entering hacking		Prior to release		*F*	*p*
		Mean	SD	Mean	SD		
Hemoglobin (g/dL)	55	13.56	0.18	10.43	0.78	82.19	<.001
PCV (%)	55	38.66	13.14	29.73	5.34	27.76	<.001
Red blood cells (10^12^/L)	55	2.55	2.53	2.59	0.93	1.88	.139
White blood cells (10^9^/L)	55	12.33	15.12	15.52	6.48	6.77	<.001
Heterophil (10^9^/L)	55	6.22	3.09	9.73	5.15	9.42	<.001
Lymphocyte (10^9^/L)	55	4.14	0.85	3.26	1.92	0.26	.854
Eosinophil (10^9^/L)	55	1.08	0.54	1.42	0.92	3.47	.020
Monocyte (10^9^/L)	55	0.64	0.24	0.71	0.47	1.60	.195
Basophil (10^9^/L)	55	0.24	11.94	0.39	0.38	2.84	.044
Thrombocytes (10^9^/L)	53	22.06	3.11	30.15	15.24	3.86	.013
Albumin (g/L)	55	13.18	0.50	14.32	1.22	20.03	<.001
Globulin (g/L)	55	17.17	1.32	18.63	3.41	6.30	<.001
Urea (mmol/L)	41	1.55	0.77	0.72	0.35	36.02	<.001
Glucose (mmol/L)	41	16.04	0.15	14.55	0.94	12.25	<.001
Cholesterol (mmol/L)	41	5.49	60.57	6.73	1.73	5.33	.002
Calcium (mmol/L)	55	2.58	574.93	2.69	0.12	15.86	<.001
AST (U/L)	55	230.07	359.90	218.85	58.77	1.51	.219
CK (U/L)	55	1086.57	188.46	1334.14	320.60	3.81	.013
Uric acid (μmol/L)	55	696.18	1.46	322.76	104.44	10.29	<.001
LDH (U/L)	55	717.31	1.18	749.36	168.36	1.89	.137
Sodium (mmol/L)	55	155.92	0.67	154.15	2.52	3.43	.022
Potassium (mmol/L)	55	2.35	1.13	3.07	0.62	4.28	.008
Body weight (kg)	53	5.13	0.92	3.92	0.68	74.53	<.001

**TABLE 3:** MANOVA analyses, effect sizes and power results of blood hematological and biochemistry parameters as dependent variables and sample, sex, and their (*) interaction as fixed factors.

	Multivariate tests of significance, effect sizes and powers							
	Test	Value	*F*	Effect df	Error df	*p*	Partial eta‐squared	Observed power (*α* = .05)
Intercept	Wilks	0.0001	158671.6	30	46	<.001	0.999	1.00
Sample	Wilks	0.0590	24.4	30	46	<.001	0.941	1.00
Sex	Wilks	0.1662	7.7	30	46	<.001	0.834	1.00
Sample × sex	Wilks	0.5553	1.2	30	46	.261	0.445	0.81

*Note*: Both sample (1 or 2, dependent on timing during hacking) and sex had a significant effect but the interaction did not.

**FIGURE 2:**
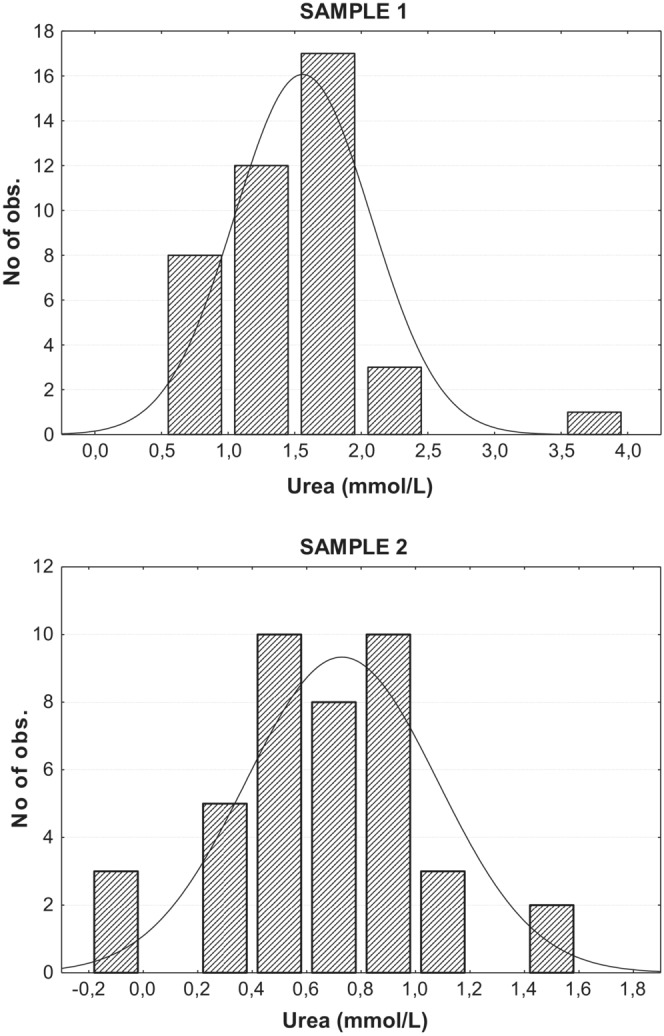
Distributions of urea values of sample 1 (soon after entering hacking) and sample 2 (just before the release from hacking). Sample 1 showed a non‐normal distribution (Shapiro–Wills = 0.889, *p* < .001) that changed to a normal distribution in sample 2 (Shapiro–Wills = 0.965, *p* = .246), after more than 1 month with ad libitum food in the hacking facility.

**FIGURE 3:**
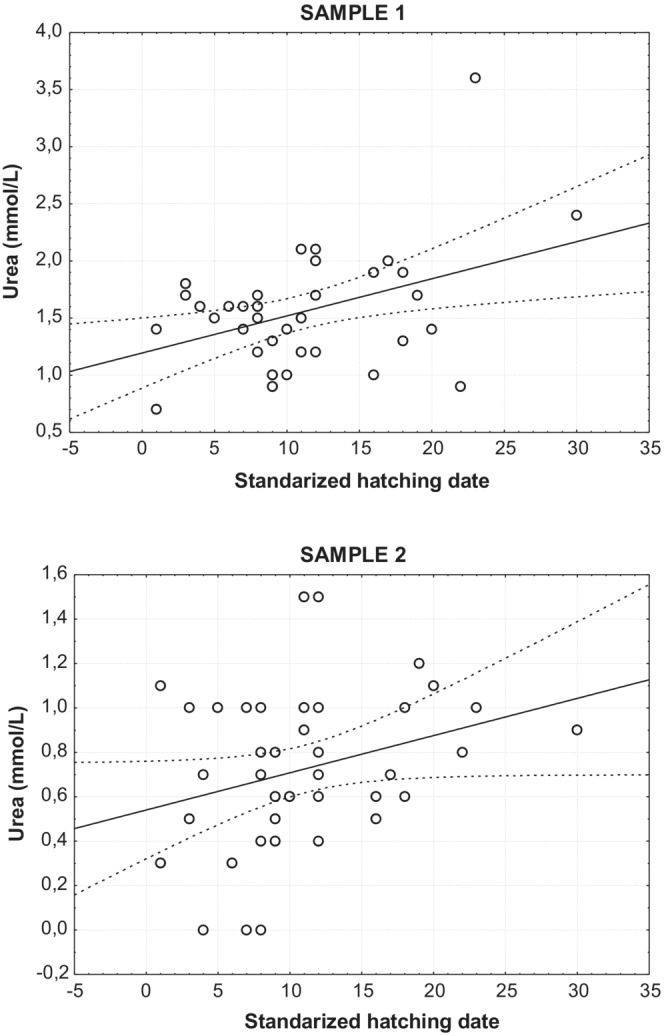
Linear regression between standardized hatching date and urea level in sample 1 (soon after entering hacking) and sample 2 (just before the release from hacking). Sample 1 showed a significant relationship with higher urea levels in later hatched birds (*r* = .397, *R* ^2^ = .16, *p* = .010). This relationship disappeared in sample 2 (*r* = .297, *R* ^2^ = .08, *p* = .059), after more than 1 month with ad libitum food in the hacking facility.

For urea, the form of the distribution also changed from an initial left‐skewed (leptokurtic) distribution in the sample when entering the hacking facility (Shapiro–Wills = 0.889, *p* < .001), to a normal distribution of values after their stay in the hacking facility (Shapiro‐Wills = 0.965, *p* = .246, Figure 2). A significant linear regression between standardized hatching date and urea level was found only for the first samples (*r* = .396, *R* ^2^ = .16, *p* = .010), but not for the second samples, after their stay in captivity (*r* = .297, *R* ^2^ = .08, *p* = .059) (Figure 3).

## 4 DISCUSSION

Our main objective was to determine if manipulation associated with hacking could have harmful consequences on health and nutritional condition of released young, after an average of 44 days of ad libitum food and no contact with parents. We found no harmful effects across many hematological and biochemical parameters. Alternatively, our results demonstrated important positive changes in health and nutritional parameters.

Body mass of nestlings varied significantly between samples, being lower in the second sample. This decrease in mass was similar for both sexes. The mass of nestling birds increases with increasing age, peaks several days prior to fledging, and then decreases until the day of fledging (Gray & Hamer, 2001; Morbey et al., 1999; Ricklefs, 1968; Sprague & Breuner, 2010). One possible function of such prefledging mass recession may be to induce fledging, i.e., nestlings lose mass because parents reduce provisioning rates to induce fledging when it is no longer beneficial for young to remain in the nest (Morbey et al., 1999). Another possible function of prefledging mass recession is that it helps optimize wing loading for newly fledged young (Morbey et al., 1999; Ricklefs, 1968; Shultz & Sydeman, 1997). Our results support the second hypothesis since food was provided ad libitum throughout the hacking period, without parental influence and any variation during the prerelease (prefledgling) period. Consequently, our results suggest a physiological mechanism, probably reducing the total amount of water in the body, as the major cause of weight recession before first flight (Ricklefs, 1968).

Hemoglobin and PCV showed a significant decrease during hacking, which indicates a diminution in the concentration of red blood cells after being fed ad libitum for more than 1 month. These results suggest a process of hemodilution associated with the ad libitum food (García‐Rodriguez, Ferrer, Carrillo, & Castroviejo, 1987), supported by the significant increase in blood proteins, globulin, and albumin. Blood proteins are essential to exert a colloidal osmotic pressure in order to aid in the preservation of blood volume and blood pH within a narrow range (Sturkie, 1976). Consequently, an increase in blood proteins cause an increase in the blood osmotic pressure, which would cause an elevation of plasma liquid, generating a temporal hemodilution effect, which induces a higher transitory blood volume (García‐Rodriguez, Ferrer, Carrillo, & Castroviejo, 1987).

Results showed a general increase in cells related with the immune system: total white blood cells, heterophils, eosinophils, and basophils. These results indicate an improvement in the immune system of young eagles after the hacking period, suggesting a general health improvement.

Results also showed that glucose levels, and glucose values decreased during hacking, which is in accordance with results for other raptor species (García‐Rodriguez, Ferrer, Carrillo, & Castroviejo, 1987). The physiological explanation for this decrease could be that in a situation of long‐term undernutrition, the glucose regulation by insulin would be relaxed. In this case, the utilization of glucose by the cells could decrease and, therefore, produce an increase in the plasma glucose (Groscolas & Rodriguez, 1981).

Cholesterol is associated with fat metabolism (Alonso‐Alvarez et al., 2003; García‐Rodriguez, Ferrer, Carrillo, & Castroviejo, 1987) and levels increased after the hacking period. Cholesterol concentrations increased in other eagles when birds were adequately fed, as also in owls, vultures, and kites (Ferrer et al., 1987). These increases in cholesterol may be merely caused by the increased lipid ingestion and absorption, and subsequent cholesterol synthesis (Ferrer & Dobado‐Berrios, 1998). In Yellow‐legged Gulls (*Larus michahellis*), cholesterol level was the strongest correlated parameter with cumulative body‐mass loss during fasting (Alonso‐Alvarez et al., 2002; Alonso‐Alvarez & Ferrer, 2001).

Creatinine kinase CK is related to muscular activity (Alonso‐Alvarez et al., 2003). Levels of plasma CK were higher toward the end of hacking. CK is found primarily in skeletal muscle, heart muscle, and brain tissue and is often used as a diagnostic indicator of muscle damage (Meredith et al., 2012). Muscle cell disruption and enzyme leakage damage can occur when muscle strength and elasticity is exceeded by exercise effort. With training, muscle cells' hypertrophy and neuromuscular coordination improves; thus, less cellular disruption occurs and trained individuals have lower CK. In Red‐tailed Hawks (*Buteo jamaicensis*), CK levels in birds that had not flown rose dramatically above baseline in the 24 h after a short period of exercise compared to a nonsignificant rise from a higher baseline in flight‐trained hawks (Knuth & Chaplin, 1994). Consequently, the rise in CK plasma levels in our WTE could be related to increasing flight exercise as they mature: increased wing flapping was observed in hacked birds in days prior to (and conditional on) their release. At the age of the first sample, the eagles in this study had never flown and, thus, it could be hypothesized that their “untrained” muscles might be more susceptible to enzyme leakage (Meredith et al., 2012).

Calcium levels vary according to ossification process (Dobado‐Berrios & Ferrer, 1997; Viñuela et al., 1991) and calcium concentration significantly increased after hacking period. This increase is to be expected in the growth of nestlings and we do not have comparable data on what may be expected for wild nestlings across the same time period.

Urea and uric acid showed a highly significant decrease after the hacking period, suggesting a decrease in catabolism rates due to ad libitum food. High values of urea have proven to be good indicators of undernourishment (Ferrer et al., 1987; García‐Rodriguez, Ferrer, Carrillo, & Castroviejo, 1987; Polo, 1995). Urea is a minor pathway for protein degradation in birds but the activity of liver arginase (the enzyme on which urea production in birds depends) increases after a prolonged fast, and hence, the rise of urea during protein catabolism may be explained by a greater arginine availability (García‐Rodriguez, Ferrer, Carrillo, & Castroviejo, 1987). Consequently, our results on urea and uric acid levels indicate improved nutritional conditions of young WTE due to hacking.

Interestingly, not only was there a change in urea concentration during hacking, but a change in the form of the distribution at the two sampling events. The first samples showed a left‐skewed distribution, with a right tail of elevated urea concentrations. Contrarily, second samples showed a normal distribution of urea values. In conclusion, the hacking period not only decreased the mean urea value but decreased the dispersion and homogenized the nutritional levels of young eagles. This effect could have interesting consequences on subsequent juvenile dispersal distances, as demonstrated in Spanish Imperial Eagles *Aquila adalberti* (Ferrer & Morandini, 2017). It has been demonstrated that maximum dispersal distances, which is critical in reintroductions actions, is positively correlated with urea level in nestlings, with those nestlings in better conditions (lower urea levels) dispersing longer distances. Those young that were fed ad libitum during hacking disperse further away and dispersal distances distribution changes to a normal one (Ferrer & Morandini, 2017).

It has been frequently found that females reproducing earlier in the season produced larger broods with offspring in better physical condition (Casado et al., 2002; Ferrer, 1994; Klomp, 1970; Moreno et al., 1997; Newton & Marquiss, 1984). For example, early hatched Spanish Imperial Eagle nestlings were better nourished than later ones (Ferrer, 1994; Muriel et al., 2015). In our study, that standardized hatching dates were only strongly correlated with the first sample but not with the second gives support to hacking improving nutritional conditions through ad libitum food, dissipating nutritional differences among donor birds from different territories. Here, we compared the birds before and after a period in captivity, while they are also undergoing a significant phase of development. That means that the potential effects of captivity cannot be separated from any effects of development and maturation. Nevertheless, study design allows to investigate whether there is evidence for negative health impacts—and results appear to point to the contrary.

In summary, it has already been established that hacking techniques, when properly applied, should not affect the subsequent dispersal behavior of released young (Ferrer & Morandini, 2017; Morandini & Ferrer, 2017a), whose survival probabilities are equal or slightly higher than wild birds (Evans et al., 2009; Muriel et al., 2020), and whose productivity in their later breeding life is equal or higher than wild birds (Morandini et al., 2017, 2019; Murgatroyd et al., 2018). Our novel study demonstrated that certain health indicators, especially those related with the immune system and nutritional parameters, also showed a significant improvement after the hacking period. Consequently, our study is consistent with others showing no harmful effects in hacking techniques that should prevent their use. This is relevant because wildlife reintroductions are a potentially important tool for conservation of endangered or threatened species, and their use is likely to increase in the future.

## AUTHOR CONTRIBUTIONS

**Miguel Ferrer:** Conceptualization (lead); formal analysis (lead). **Rhian Evans:** Data curation (equal); writing – review and editing (equal). **Joanna Hedley:** Data curation (equal); investigation (equal). **Simon Hollamby:** Data curation (equal); methodology (equal); writing – review and editing (equal). **Anna Meredith:** Conceptualization (equal); data curation (equal); investigation (equal). **Virginia Morandini:** Conceptualization (equal); formal analysis (supporting); investigation (supporting); methodology (equal); supervision (equal); validation (equal); writing – review and editing (equal). **Owen Selly:** Data curation (equal); writing – review and editing (equal). **Claire Smith:** Data curation (equal). **D. Phil Whitfield:** Conceptualization (lead); data curation (lead); formal analysis (equal); funding acquisition (lead); investigation (equal); methodology (lead); supervision (equal); validation (equal); writing – review and editing (equal).

## Data Availability

The raw data supporting the conclusions of this article will be made available at the CSIC public repository: http://hdl.handle.net/.
